# Brain tumor classification from FFPE samples using nanopore methylation sequencing

**DOI:** 10.1093/narcan/zcaf038

**Published:** 2025-10-30

**Authors:** Galina Feinberg-Gorenshtein, Assaf Grunwald, Carlo Vermeulen, Nurit Gal Mark, Elena Shinderman-Maman, Adva Levy-Barda, Keren Shichrur, Michal Hameiri-Grossman, Orli Michaeli, Shira Amar, Suzanna Fichman, Abraham Natan, Tali Siegal, Shlomit Yust-Katz, Hanna Weiss, Osnat Konen, Amir Kershenovich, Andrew A Kanner, Jeroen de Ridder, Helen Toledano, Shai Izraeli, Yehudit Birger, Yuval Ebenstein

**Affiliations:** The Rina Zaizov Division of Pediatric Hematology-Oncology, Schneider Children’s Medical Center of Israel, P.O. Box 559, Petach Tikva, Israel; Department of Physical Chemistry, School of Chemistry, Tel Aviv University, P.O. Box 39040, Tel Aviv, Israel; Center for Molecular Medicine, University Medical Center Utrecht, P.O. Box 85500, 3508 GA Utrecht, The Netherlands; The Rina Zaizov Division of Pediatric Hematology-Oncology, Schneider Children’s Medical Center of Israel, P.O. Box 559, Petach Tikva, Israel; Felsenstein Medical Research Center, Tel Aviv University, Rabin Medical Center (Beilinson Campus), 39 Jabotinsky Street, Petah Tikva 4941492, Israel; The Rina Zaizov Division of Pediatric Hematology-Oncology, Schneider Children’s Medical Center of Israel, P.O. Box 559, Petach Tikva, Israel; Biobank, Department of Pathology, Rabin Medical Center (Beilinson Hospital), Attn: Biobank, 39 Ze’ev Jabotinsky Street, Petah Tikva 4941492, Israel; Department of Digital Medical Technologies, Holon Institute of Technology, P.O. Box 305, Holon, Israel; The Rina Zaizov Division of Pediatric Hematology-Oncology, Schneider Children’s Medical Center of Israel, P.O. Box 559, Petach Tikva, Israel; The Rina Zaizov Division of Pediatric Hematology-Oncology, Schneider Children’s Medical Center of Israel, P.O. Box 559, Petach Tikva, Israel; The Rina Zaizov Division of Pediatric Hematology-Oncology, Schneider Children’s Medical Center of Israel, P.O. Box 559, Petach Tikva, Israel; Tel Aviv University, Gray Faculty of Medical and Health Sciences, P.O. Box 39040, Tel Aviv, Israel; The Rina Zaizov Division of Pediatric Hematology-Oncology, Schneider Children’s Medical Center of Israel, P.O. Box 559, Petach Tikva, Israel; Department of Pathology, Beilinson Hospital Institute of Pathology, 39 Ze’ev Jabotinsky Street, Petah Tikva 4941492, Israel; Department of Pathology, Beilinson Hospital Institute of Pathology, 39 Ze’ev Jabotinsky Street, Petah Tikva 4941492, Israel; Tel Aviv University, Gray Faculty of Medical and Health Sciences, P.O. Box 39040, Tel Aviv, Israel; Neuro-Oncology Unit, Davidoff Cancer Center at Rabin Medical Center, 39 Ze’ev Jabotinsky Street, Petah Tikva 4941492, Israel; Tel Aviv University, Gray Faculty of Medical and Health Sciences, P.O. Box 39040, Tel Aviv, Israel; Neuro-Oncology Unit, Davidoff Cancer Center at Rabin Medical Center, 39 Ze’ev Jabotinsky Street, Petah Tikva 4941492, Israel; Department of Pathology, Beilinson Hospital Institute of Pathology, 39 Ze’ev Jabotinsky Street, Petah Tikva 4941492, Israel; The Institute of Imaging, Schneider Children’s Medical Center of Israel, P.O. Box 559, Petach Tikva, Israel; Division of Pediatric Neurosurgery, Schneider Children’s Medical Center of Israel, P.O. Box 559, Petach Tikva, Israel; Department of Pathology, Beilinson Hospital Institute of Pathology, 39 Ze’ev Jabotinsky Street, Petah Tikva 4941492, Israel; Department of Neurosurgery, Rabin Medical Center, 39 Ze’ev Jabotinsky Street, Petah Tikva 4941492, Israel; Center for Molecular Medicine, University Medical Center Utrecht, P.O. Box 85500, 3508 GA Utrecht, The Netherlands; The Rina Zaizov Division of Pediatric Hematology-Oncology, Schneider Children’s Medical Center of Israel, P.O. Box 559, Petach Tikva, Israel; Tel Aviv University, Gray Faculty of Medical and Health Sciences, P.O. Box 39040, Tel Aviv, Israel; The Rina Zaizov Division of Pediatric Hematology-Oncology, Schneider Children’s Medical Center of Israel, P.O. Box 559, Petach Tikva, Israel; Felsenstein Medical Research Center, Tel Aviv University, Rabin Medical Center (Beilinson Campus), 39 Jabotinsky Street, Petah Tikva 4941492, Israel; The Rina Zaizov Division of Pediatric Hematology-Oncology, Schneider Children’s Medical Center of Israel, P.O. Box 559, Petach Tikva, Israel; Felsenstein Medical Research Center, Tel Aviv University, Rabin Medical Center (Beilinson Campus), 39 Jabotinsky Street, Petah Tikva 4941492, Israel; Department of Physical Chemistry, School of Chemistry, Tel Aviv University, P.O. Box 39040, Tel Aviv, Israel; Sagol School of Neuroscience, Tel Aviv University, P.O. Box 39040, Tel Aviv-Yafo, Israel

## Abstract

Oxford Nanopore Technology (ONT)-based methylation sequencing is emerging as a powerful approach for the rapid and accurate classification of brain tumors, an essential component of precision oncology. However, its broader clinical adoption has been limited by reliance on fresh-frozen (FF) tissue, whereas the vast majority of clinical specimens are formalin-fixed paraffin-embedded (FFPE). In this study, we address this limitation by evaluating the effects of FFPE processing on DNA methylation profiles and introducing a validated protocol for ONT-based classification using DNA extracted directly from pathology-marked regions on stained FFPE slides. This approach enables the targeted selection of tumor-rich areas following histological assessment, thereby improving DNA input quality and tumor content. We demonstrate that even small, low-input samples (≥25 ng) can be successfully classified using this method, with high concordance to final integrated neuropathological diagnoses. Our results show that, despite modest methylation loss associated with formalin fixation, classification performance remains robust. Notably, we identify a correlation between methylation degradation and fixation time, supporting a recommendation to limit formalin exposure to ≤3–4 days when possible. By enabling accurate methylation-based tumor classification from routinely processed, stained FFPE tissue, our protocol integrates seamlessly into existing clinical workflows. This expands the accessibility of ONT-based diagnostics and supports informed, timely treatment decisions—even in cases with minimal tissue availability or urgent clinical need.

## Graphical Abstract

**Graphical Abstract:**
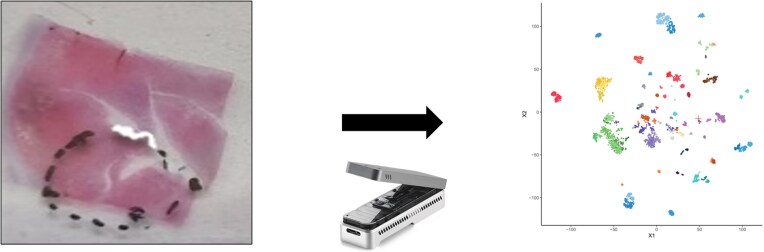

## Introduction

Accurate classification of tumor types and subtypes is crucial for ensuring precise and comprehensive patient treatment [1, 2]. For instance, the World Health Organization (WHO) classification of central nervous system (CNS) tumors encompasses over 10 major tumor classes, each comprising numerous subclasses, totaling more than a hundred distinct entities that require diverse treatments and are associated with different prognosis and clinical courses [3, 4]. These tumor entities frequently exhibit resemblances in morphological, spatial, and genetic characteristics [5], rendering their diagnosis challenging. Moreover, neuropathologists may provide varying interpretations of histopathological results, adding subjectivity to the process [6, 7]. Epigenetics, on the other hand, and particularly DNA methylation, has proven to be a robust and stable biomarker for accurately distinguishing the vast majority of these tumor subtypes [1]. Hence, methylation-based classification has been incorporated into the 2021 WHO classification of CNS tumors [3]. Consequently, the examination of tumor methylation patterns has recently become a part of clinical diagnostic procedures in some centers, with methylation-based classifiers already available for CNS tumors and sarcomas, with other classifiers under development for different tumor types [8].

Yet, the incorporation of DNA methylation into the diagnostic workup of brain tumors has proved challenging. The Infinium MethylationEPIC (EPIC) array remains the gold standard for CNS methylation-based profiling due to its robustness and genome-wide coverage [9]. However, it also presents practical limitations, including relatively high costs, long turnaround times, and the need to batch multiple samples. Although recent studies have demonstrated successful application with DNA inputs as low as 100 ng [10, 11], many clinical workflows continue to favor higher input amounts to reduce the risk of sample dropout and ensure consistent data quality. In addition, the assay involves bisulfite conversion and polymerase chain reaction (PCR) amplification, which are known DNA to introduce systematic biases. To overcome these challenges, recent studies have successfully employed Oxford Nanopore Technology (ONT), which enables rapid, direct epigenetic sequencing from native DNA, thus eliminating the need for PCR amplification or bisulfite conversion, both of which can contribute to technical bias. Consequently, several healthcare centers have begun incorporating ONT-based workflows into their diagnostic pipelines [12–15].

Nevertheless, the widespread adoption of ONT for tumor classification and other biopsy-derived DNA analyses face a major obstacle: the platform preferably requires concentrated, unamplified DNA of high purity and high molecular weight—typically obtainable only from fresh frozen (FF) biopsy samples. In contrast, most health centers predominantly provide formalin-fixed paraffin-embedded (FFPE) tissue due to logistical constraints in sample transport and storage [7, 16].

Additionally, the unknown tumor fraction (TF) in FF samples introduces uncertainty, as a low proportion of tumor cells in the homogenized tissue can impair both classification accuracy and the associated confidence score [17].

In FFPE samples on the other hand, histopathological staining can be performed so that TF is not only known but can be selectively enriched for analysis [18].

In a recent study, Afflerbach *et al.* [19] demonstrated that ONT-based classification from FFPE tissues derived DNA closely aligns with classifications obtained via EPIC arrays. Building on this, we present an approach for pre-extraction enrichment of tumor content for ONT sequencing and methylation classification. FFPE DNA is extracted after histological staining, enabling targeted selection of tumor-rich regions identified by a pathologist. This approach helps mitigate the effects of intratumoral heterogeneity, a well-recognized challenge in solid tumor profiling [5], and significantly improves the yield and quality of tumor-derived DNA for downstream analysis.

Importantly, we show that this method is effective even when applied to small regions yielding limited DNA quantities. We directly compare all ONT-based classifications to the final integrated neuropathological diagnosis, observing high concordance. This included a combination of histopathological evaluation and supporting molecular data derived from molecular orthogonal methods, including Next-Generation Sequencing (NGS), Fluorescence in situ Hybridization (FISH), Nanostring, and EPIC methylation arrays. This approach more accurately reflects real-world diagnostic workflows.

Our protocol reduces DNA input requirements from the hundreds of nanograms used in prior ONT-based classification studies to as little as 25 ng. Additionally, we performed targeted analyses on matched FF and FFPE sample pairs to assess fixation-related effects on ONT-derived methylation profiles.

This methodology was applied across 30 cases spanning 20 molecular subgroups, including rare and diagnostically challenging tumor types. By integrating pathological guidance with ONT sequencing and classification, our approach supports real-world implementation of low-input, FFPE-based tumor methylation profiling.

## Materials and methods

### FFPE slide generation and TF assessment via standard histology

FFPE tissue fixation, processing, and embedding were done using standard protocols. Briefly, tissues were stored in 4% buffered formalin immediately after removal by the surgeon. The tissues typically remained in formalin for 1–5 days before the tumor region was sectioned by a neuropathologist. The tissues were then processed automatically using the Tissue-Tek VIP 6 (Model VIP 6-E2), followed by embedding in paraffin (wax) to form blocks. Finally, the blocks were cut into 4–5 μm unstained sections and placed on slides for staining. Post Hematoxylin and Eosin (H&E staining) (following standard protocols), neuropathologists marked selected regions enriched for tumor content.

### DNA extraction and quantification

DNA library preparation for Oxford Nanopore sequencing requires high molecular weight DNA in relatively high amounts, concentrations, and purity—criteria that are often difficult to meet with FFPE-derived material. To address these challenges, we implemented specific modifications to the DNA extraction and library preparation protocols, with a particular focus on accommodating the limitations of low-input FFPE samples:

DNA was isolated using either the QIAamp DNA FFPE Tissue Kit (Qiagen, Hilden, Germany) or the RecoverAll Multi-Sample RNA/DNA Kit (DNA component only, Invitrogen, Thermo Fisher, US), with the following modifications:

Instead of using xylene, tissue sections from 7–17 slides were pooled into a 1.5 ml tube containing 400 μl of digestion buffer. The tube was heated at 90°C for 3 min, then centrifuged at 14 000 × *g* for 1 min. A brief incubation on ice allowed the paraffin to solidify as a ring, which was then manually removed. This optimized heat-based deparaffinization protocol, routinely used in our clinical molecular oncology lab, reduces toxicity, simplifies handling, and ensures effective paraffin removal without compromising DNA recovery.

DNA from FF tumor tissue was extracted using the QIAamp DNA Micro Kit (Qiagen, Hilden, Germany) according to the manufacturer’s instructions. We observed reduced sequencing output from FFPE-derived libraries compared to matched FF samples, as illustrated in Supplementary Fig. S2.

DNA concentrations were measured using a Qubit Flex fluorometer with the double-stranded DNA HS Assay Kit (Invitrogen, Thermo Fisher, US). DNA purity was assessed using a NanoDrop spectrophotometer (Invitrogen, Thermo Fisher, US) by measuring absorbance at 260/280 nm.

### Library preparation and nanopore sequencing

Library preparation for FFPE-derived DNA was performed using the Ligation Sequencing Kit V14 (SQK-LSK114, ONT, UK) with specific modifications to optimize performance on low-input and low molecular weight DNA. These optimizations were adapted from approaches used for cfDNA sequencing, given the similar challenges [6, 20, 21].

In the DNA repair and end-preparation step, incubation times were extended to 30 min at 20°C followed by 30 min at 65°C. This adjustment was necessary to improve the efficiency of enzymatic repair in FFPE-derived DNA, which may contain lesions or fragmentation due to formalin-induced crosslinking and degradation.

The bead-to-sample ratio was increased during purification steps to enhance DNA recovery and clean-up efficiency. Specifically, 180 μl of beads were used in the DNA repair and end-prep step, and 120 μl in the adapter ligation and clean-up step. These increases improved binding kinetics and DNA capture, facilitating higher overall yield and cleaner libraries.

In the adapter ligation step, the ligation incubation was extended to 40 min to improve adapter attachment efficiency. The final elution volume was reduced to 12 μl to concentrate the library, enabling sufficient input for sequencing even from low-yield FFPE extractions.

This protocol was validated for FFPE blocks stored at −20°C up to 72 months before sequencing.

### Data processing and CNS classification

Raw POD5 files were basecalled using Guppy v6.4.6 (ONT, UK) with the model configuration dna_r10.4.1_e8.2_400bps_modbases_5mc_cg_hac_mk1c.cfg. Basecalled reads were aligned to the T2T-CHM13 reference genome using minimap2 v2.24 [22]. The resulting BAM files were merged, sorted, and indexed using samtools v1.16.1 [23], and subsequently analyzed using the Sturgeon classifier [12] under default parameters for CNS tumor classification. Sturgeon-based classification results and scores form the basis for method validation and subsequent analyses throughout this study.

In addition, we tested CNS classification using the NanoDx framework [15, 24] which requires a distinct post-processing pipeline. For this analysis, POD5 files were basecalled using Dorado v0.3.4 (ONT, UK) with the model dna_r10.4.1_e8.2_400bps_hac@v4.2.0, using the –modified-bases 5mCG_5hmCG flag to enable direct detection of cytosine modifications. Reads were aligned to the hg38 reference genome using minimap2 v2.24 [22], and CpG methylation levels were extracted using modkit v0.1.12 with the pileup command. The resulting data were provided as input to the NanoDx classifier.

Results obtained from both classification frameworks are summarized in Supplementary Table S2.

All analyses were performed on a Linux-based system (Ubuntu 22.04.3) equipped with an NVIDIA GeForce RTX 3060 GPU.

To generate methylation scores per 4mer an in-house Python script was used to extract the locations of all possible 4-mer combinations for the human genome reference in BED format. The resulting BED files were intersected with the output files from the modkit pileup to generate a location and score file for each sample and 4-mer (using bedtools V2.30.08). Methylation values were averaged for each 4-mer in each file, and methylation ratios were calculated for each observation.

## Results

**Figure 1:**
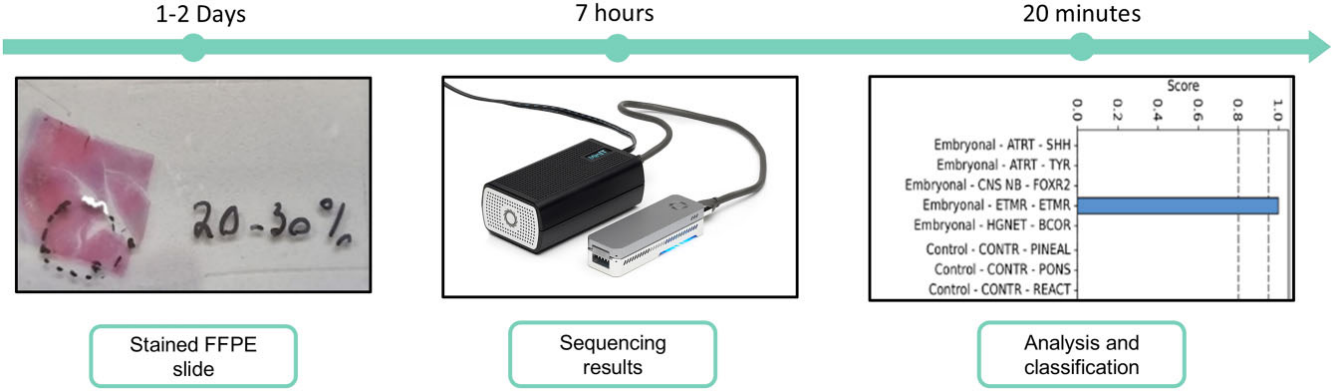
Schematic representation of the typical timeline and steps of our suggested protocol—from biopsy to classification: Left: Generation of FFPE slides with histochemical staining typically takes 1–2 days. DNA for classification is purified only from regions marked by the pathologist. Middle: Our adjusted protocol for DNA purification from histological slides takes ∼4 h followed by roughly 90 min for adjusted ONT DNA library preparation and ∼120 min of sequencing are required to gather a sufficient amount of data for classification (∼7 h all together). Right: “Sturgeon” classification of sequencing data takes ∼20 min on a local computer.

The conceptual framework and timeline of the workflow are presented in Fig. 1. As an illustrative example, we demonstrate how histopathological staining of a low TF sample can guide selective DNA extraction from regions with relatively higher tumor content (80%–90%). This enables focused downstream analysis despite the overall low tumor burden. Successful classification in such a scenario highlights the potential utility of histology-guided sequencing in clinically challenging cases (see the ‘Materials and methods’ section for adjusted protocol for DNA extraction and sequencing from FFPE samples).

**Figure 2:**
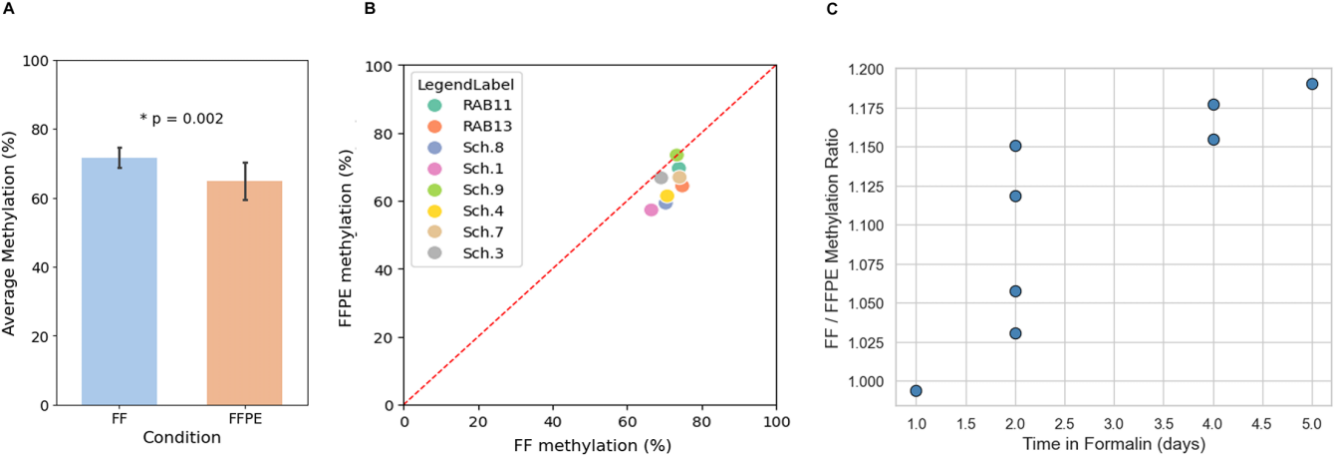
(**A**) Mean genome-wide CpG methylation percentages in FF (blue) and FFPE (orange) DNA across eight matched sample pairs. A paired two-tailed *t*-test demonstrates a significant reduction in FFPE DNA (*P* = 0.002). (**B**) Per-patient comparison of average methylation in FF (x-axis) versus FFPE (y-axis) DNA derived from the same tumour. Colors correspond to individual samples patients for whom matched FF and FFPE samples were available (see inset legend). Dashed red line represents the diagonal. (**C**) Relationship between time in formalin and methylation preservation. Each point represents the ratio of methylation levels in FF versus FFPE tissue samples, plotted against the number of days the FFPE sample spent in formalin prior embedding.

To generalize this approach, we first set out to verify that the FFPE process does not hamper DNA methylation and hence the methylation-based classification. Tumor samples were collected from eight patients and processed as both FFPE and FF. Samples were subjected to DNA extraction and methylation sequencing (Supplementary Table S3). We observed a significant global reduction in methylation levels in FFPE samples compared to their matched fresh-frozen (FF) counterparts (Fig. 2A) consistent with the lower values seen across individual CpG sites (Supplementary Fig. S1). Across the cohort, average methylation levels decreased from 71.5% in FF samples to 64.5% in FFPE samples (*P* = 0.002, paired *t*-test). This reduction was consistent across individual patient pairs (Fig. 2B) and appeared to correlate with the duration of formalin fixation. This can be explained by deamination of both cytosine and 5-methylcytosine induced by formalin. However, deamination rate is substantially higher for mC compared to C leading to preferential loss of methylated cytosines (Fig. 2C; see Supplementary Information for further discussion of potential biochemical mechanisms and technical considerations).

Although modest in magnitude, this global reduction raised the question of whether FFPE processing introduces systematic biases that could affect methylation-based analyses. Specifically, we considered whether the observed methylation loss was due to random demethylation or reflected sequence-specific effects that might interfere with downstream applications, such as CNS tumor classification.

To address this, CpG sites were categorized by their surrounding 4-mer sequence context, defined by the two nucleotides flanking each side of the CpG (e.g. ACGA, ACGC), resulting in 16 canonical sequence combinations. The relationship between each 4-mer context and the Δ value—representing methylation loss in FFPE relative to FF samples (Supplementary Fig. S5) was then evaluated.

Comparative modeling demonstrated that the majority of the Δ value variance is explained by baseline methylation differences associated with each sequence context, rather than alterations introduced by FFPE processing (see Supplementary Information). Consequently, FFPE processing does not introduce significant sequence-specific biases, and methylation profiles from these samples remain appropriate for downstream analyses. In addition it should be noted that differences in tumor content between FFPE and FF samples may also contribute to the observed methylation differences. Importantly, as we demonstrate below, classification performance remained robust across FFPE samples, underscoring the reliability of nanopore-based methylation profiling even in the presence of modest global methylation loss.

Next, given that many times the region identified as high TF is relatively small (Fig. 1) allowing to extract only low amounts of DNA, we wished to assess the lower limit of DNA input amount for classification. Purified DNA from three previously classified FFPE samples were analyzed with varying input amounts of extracted DNA, ranging from 1000 to 25 ng. We performed methylation sequencing and classification for all samples and obtained correct classification even for the lowest input amount of 25 ng DNA (Supplementary Table S1; see the ‘Materials and methods’ section for adjusted FFPE protocol). In addition, correct classification was achieved even for cases where <5% of the sample was marked by the pathologist or for samples retrieved by needle biopsies. This underscores the reliability and precision of the methodology in clinical settings.

To validate the clinical applicability of FFPE samples for methylation-based classification, we retrospectively sequenced 30 DNA samples purified from FFPE CNS tumor tissues of 13 pediatric and 17 adult patients using ONT methylation sequencing.

For classification, we used two publicly available pipelines: NanoDx (version: v0.6.2) [13] and Sturgeon (v0.3.4) [12]. The latter demonstrated better performance and achieved high concordance with final neuropathological evaluations supported by molecular test results. Consequently, the results presented here are based on the Surgeon classifier (classification outcomes and confidence scores for both methods are provided in Supplementary Table S2). In all cases included in our cohort, the final integrated neuropathological diagnosis was established through a combination of histopathological evaluation and supporting molecular tests performed by orthogonal methods. Comprehensive results, including patient information, sample details, and sequencing metrics, are provided in Supplementary Table S2. The Sturgeon classifier uses two interpretation thresholds to guide diagnostic confidence. A calibrated score above 0.95 is considered a confident diagnosis, indicating a high-probability match to a reference methylation class. Scores between 0.80 and 0.95 are interpreted as a likely diagnosis, where the classification is plausible but may benefit from additional validation, such as further sequencing. Scores below 0.80 are generally regarded as unreliable [13].

Classification performance with the Sturgeon classifier is summarized below:

Thirteen cases (∼44%) were classified with high confidence scores (≥0.95), all of which were in full concordance with final integrated neuropathological diagnosis.

Six cases (20%) were classified with confidence scores between 0.90 and 0.95. All were concordant with final neuropathological evaluations. Notably, patient RAB.13 was classified by methylation profiling *as Glioblastoma, IDH wildtype, subclass RTK I*, while histopathological evaluation identified the case as a *gliosarcoma*. This does not represent a discrepancy but rather reflects a distinction between histology-based and methylation-based classification: *gliosarcoma* is a histological variant of *Glioblastoma, IDH wildtype*, and is not represented as a distinct methylation class. In the reference dataset [1], gliosarcoma cases are typically assigned to existing *Glioblastoma, IDH wildtype subclasses*, including RTK I.

Seven cases (23%) were classified with confidence scores between 0.80 and 0.90. Of these, five were concordant with final neuro-pathology diagnosis. Two cases showed discordances: one (Sch.12) was classified as control tissue, which would not have altered patient management. Additional case (RAB6 sample with TF ∼60%) yielded a methylation-based classification of IDH glioma, subclass astrocytoma (calibrated score = 0.87), whereas the final integrated neuropathological diagnosis was oligodendroglioma, IDH-mutant and 1p/19q-codeleted, WHO grade 2. This discrepancy may stem from the limitation of current methylation classifiers in distinguishing between IDH-mutant astrocytomas and oligodendrogliomas, particularly in cases where methylation profiles are borderline or atypical. Although the classifier robustly captured the IDH-mutant signature, it failed to reflect the co-deletion of 1p/19q, which was confirmed by NGS and FISH (Supplementary Table S2). The remaining four cases (13%) had confidence scores below 0.80. Three of these were concordant with integrated neuro-pathological evaluations, and one showed disagreement. The classifier assigned the sample (RAB.12) to the *IDH glioma, subclass astrocytoma* class with a sub-threshold calibrated score of 0.68. In contrast, the final diagnosis was *glioblastoma, IDH-wildtype, CNS WHO grade 4*.

These findings underscore the importance of interpreting classifier results in the context of confidence scores and integrating them with histological and molecular data, particularly in challenging or borderline cases. The overall good classification performance emphasizes the robustness of the classifier across a range of tumor types and confidence levels, supporting its integration into diagnostic workflows. Notably, our study expands upon previous work [19] by capturing a broader spectrum of CNS tumors across 20 molecular subgroups. Rare entities such as embryonal tumor with multilayered rosettes (ETMR), Schwannoma, and CNS high-grade neuroepithelial tumors with BCOR alteration were all correctly classified, demonstrating the feasibility of using ONT methylation sequencing to detect diagnostically challenging subtypes and enhancing its clinical utility.

To illustrate the real-world diagnostic value of this approach, we present three representative pediatric cases where ONT-based methylation profiling significantly contributed to clinical decision-making:

**Case A (Patient Sch.3):** A 2.2-year-old male presented with right hemiparesis. Magnetic resonance imaging (MRI) revealed a large left parietal-temporal tumor with a large metastasis in the suprasellar region behind the optic chiasm and suspected leptomeningeal spread (Supplementary Fig. S4A, a and b). The patient initially received upfront chemotherapy at another center without biopsy, but experienced clinical and radiographic deterioration. At Schneider Children’s Medical Center, gross total resection of the temporal lesion was performed. Initial histopathological analysis of an FFPE biopsy indicated a malignant tumor, not otherwise specified (NOS), with a prolonged turnaround time for the final diagnosis. However, ONT-based methylation profiling rapidly and confidently classified the tumor as “Meningioma”. Twenty days later, histopathological examination confirmed an atypical meningioma, WHO grade 2, associated with meningioangiomatosis.

The rapid turnaround was critical; 4 days postoperatively, the patient developed sudden blindness. The classification as meningioma guided treatment, as these tumors are resistant to chemotherapy and radiotherapy, it was avoided due to the patient’s young age. Instead, debulking of the suprasellar tumor was performed to relieve chiasmal compression, followed by treatment with bevacizumab, resulting in tumor stabilization and restoration of vision.

**Case B (Patient Sch.1)**: A 5.5-year-old female presented with vomiting and left facial nerve palsy. MRI demonstrated a heterogeneous mass in the left cerebellopontine angle (Supplementary Fig. S4B, a). Subtotal resection was performed, and initial H&E staining of biopsy suggested an atypical teratoid/rhabdoid tumor (Supplementary Fig. S4B, b). However, INI1 (SMARCB1) immunostaining showed a mosaic pattern (Supplementary Fig. S4B, c), precluding a definitive diagnosis. Chromosomal microarray analysis (CMA) (Cytoscan HD, Thermo Fisher Scientific) revealed monoallelic loss of SMARCB1, and an NGS panel (Oncomine Childhood Cancer Research Assay; Thermo Fisher Scientific) did not identify pathogenic variants in SMARCB1 or SMARCA4. Nevertheless, our sequencing protocol on FFPE tissue allowed us to classify the tumor as ATRT, MYC subgroup, resulting in the initiation of appropriate chemotherapy. This diagnosis was subsequently reconfirmed a few weeks later by Infinium Methylation EPIC array (Brain tumor classifier; Version: 11b4).

**Figure 3:**
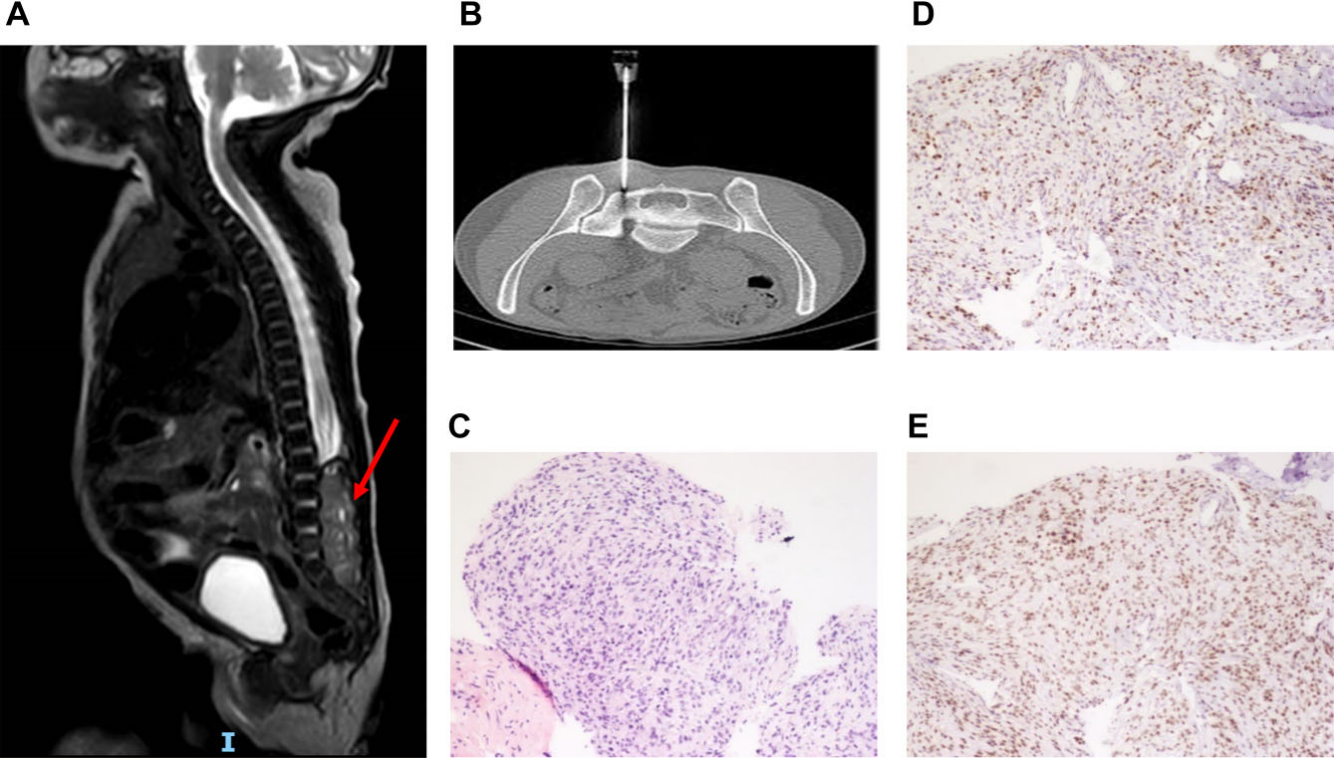
Patient Sch.9: (**A**) MRI shows tumor filling the spinal canal at the level of L2-S2, T2 sagittal view. Tumor showed heterogeneous enhancement post contrast. (**B**) CT-guided needle biopsy provided only 120 ng of DNA of poor quality. Subsequent open biopsy confirmed the ONT based diagnosis. (**C**) H&E staining of Atypical teratoid rhabdoid tumor, WHO grade 4. (**D**) SMARCB1 (INI1) immunohistochemistry staining showing nuclear loss of expression in tumor cells and retained expression in endothelial cells. (**E**) BRG1 immunohistochemistry staining is retained.

**Case C (Patient Sch.9)**: A female preterm infant was delivered at 33 + 4 weeks’ gestation via emergency cesarean section due to prenatal ultrasound findings of an enlarged bladder and bilateral hydronephrosis. Postnatal MRI revealed a spinal cord lesion extending from L2 to S2 (Fig. 3A). Neuroblastoma was initially suspected based on clinical and imaging findings. A Computed Tomography (CT) guided biopsy (Fig. 3B), yielded only a small FFPE sample due to challenging tumor location and the infant’s low weight.

Because of low tissue amounts (120 ng DNA) and poor DNA quality, both histopathology and NGS panel analyses were inconclusive. However, ONT-based methylation profiling classified the tumor as ATRT, subclass MYC subgroup. Age-adapted chemotherapy following the EuRhab protocol was initiated. A subsequent open biopsy confirmed the diagnosis; pathology immunostaining strongly suggested ATRT (Fig. 3C–E), and both CMA (Cytoscan HD, Thermo Fisher Scientific) and NGS panel analysis demonstrated a bi-allelic SMARCB1 deletion, corroborating the molecular diagnosis [25].

## Discussion

This study demonstrates the clinical utility and technical feasibility of ONT-based methylation profiling for CNS tumor classification using FFPE tissue, including DNA extracted directly from histologically stained slides. By integrating pathologist-guided selection of tumor-rich regions, we achieved accurate classification even in diagnostically challenging scenarios, including cases with limited tumor material, needle biopsies, or ambiguous histology. In three representative pediatric cases, ONT-based profiling directly contributed to timely diagnostic resolution and informed patient management, highlighting its potential as an early, actionable diagnostic tool. Our optimized protocol enabled robust classification from as little as 25 ng of input DNA and supported additional analyses, including copy number variation (CNV) profiling (Supplementary Fig. S3), underscoring the method’s flexibility and practical relevance for routine clinical implementation.

Previous studies [20] utilized 400 ng of input DNA, such amounts are often difficult to obtain from FFPE-derived CNS tumors due to tissue limitations. The ability to achieve high-confidence classification with as little as 25 ng of input DNA, may be decisive in determining whether a classification can be made at all, especially in low-yield or diagnostically ambiguous cases. This highlights a key practical advantage of our approach and further supports the integration of ONT methylation profiling into standard diagnostic workflows.

Despite this success, several challenges must be addressed before widespread clinical adoption. These include the need for formal regulatory approval, multi-institutional validation, and standardized protocols to ensure reproducibility. Additionally, a subset of samples yielded confidence scores below diagnostic thresholds, reinforcing the importance of interpreting ONT-based classifications alongside histopathological and molecular data.

Our matched FF and FFPE analysis further revealed a modest but consistent reduction in global methylation in FFPE samples. While this did not impair classification, we observed a correlation with fixation time. As such, we recommend limiting formalin exposure to 3–4 days when FFPE samples are intended for methylation analysis. Further research is needed to define fixation thresholds more precisely and to elucidate the broader impact of FFPE processing on epigenetic integrity.

Looking ahead, the potential of ONT-based diagnostics extends beyond classification alone. While the MinION platform used here provided sufficient coverage for methylation and CNV analysis, deeper sequencing on high-throughput systems such as the PromethION may enable comprehensive molecular profiling—including detection of somatic mutations, structural variants, and epigenetic signatures—in a single assay [26]. With continued protocol refinement, broader clinical validation, and integration into diagnostic pipelines, ONT methylation profiling may offer a scalable, cost-effective, and rapid alternative to current gold-standard approaches in precision neuro-oncology.

### Ethics statement

The study was conducted in accordance with the Declaration of Helsinki and adhered to local guidelines and regulations. Ethical approval was obtained from the Institutional Review Board (IRB) of Rabin Medical Center (RMC), under approvals IRB #0012-08-RMC and 0039-17-RMC. Written or electronic informed consent was obtained from all participants or their legal representatives, including consent for publication of study results, under approval number 920061517.

## Supplementary Material

zcaf038_Supplemental_Files

## Data Availability

The sequencing data generated in this study have been deposited in the NCBI Sequence Read Archive (SRA) under the accession number PRJNA1146547. Additional data supporting the findings of this study are available upon reasonable request from the corresponding author.
