# Supplementary material for: Brain tumor classification from FFPE samples using nanopore methylation sequencing
Source: NAR Cancer. 2025 Oct 30;7(4):zcaf038. doi: 10.1093/narcan/zcaf038 (PMC12574666; doi:10.1093/narcan/zcaf038)
Supplement: zcaf038_Supplemental_Files [file zcaf038_supplemental_files.zip › Supplementary Tables legends.docx]

**Supplementary Table 2.** Summary of cohort metadata, sequencing run metrics, and tumor-methylation classification results. The table compiles patient/sample characteristics alongside outputs and calibrated confidence scores from the Sturgeon and NanoDx classifiers; interpretation thresholds are detailed in the Methods. “NA” indicates data not available.

**Supplementary Table 3.** Summary of per patient paired fresh-frozen (FF) and FFPE, including sample-level metadata, sequencing run metrics, and tumor-methylation classification results for both sample types. “NA” indicates not available.
