# Supplementary material for: Brain tumor classification from FFPE samples using nanopore methylation sequencing
Source: NAR Cancer. 2025 Oct 30;7(4):zcaf038. doi: 10.1093/narcan/zcaf038 (PMC12574666; doi:10.1093/narcan/zcaf038)
Supplement: zcaf038_Supplemental_Files [file zcaf038_supplemental_files.zip › Supplementary_Information_Clean.docx]

**Brain Tumor Classification from FFPE Samples Using Nanopore Methylation**

**Sequencing - Supplementary Information**

**Authors**

Galina Feinberg-Gorenshtein^1†^, Assaf Grunwald^2*†^, Carlo Vermeulen^4^, Nurit Gal Mark^1,6^, Elena Shinderman-Maman^1^, Adva Levy-Barda^7,7,8^, Keren Shichrur^1^, Michal Hameiri-Grossman^1^, Orli Michaeli^1,5^, Shira Amar^1^, Suzanna Fichman^9^, Abraham Natan^9^, Tali Siegal^5,10^, Shlomit Yust-Katz^5,10^, [Hanna Weiss](https://pubmed.ncbi.nlm.nih.gov/?term=Weiss+H&cauthor_id=17685476)^9^, Osnat Konen^11^, Amir  Kershenovich^12^, Andrew A. Kanner^13^, Jeroen de Ridder^4^, Helen Toledano^1,5^, Shai Izraeli^1,6^, Yehudit Birger^1,6*^ and [Yuval Ebenstein](https://pubmed.ncbi.nlm.nih.gov/?term=Ebenstein+Y&cauthor_id=30485249)^2,3*^.

**Affiliations**

1. The Rina Zaizov Division of Pediatric Hematology-Oncology, Schneider Children’s Medical Center of Israel, Petach Tikva, Israel
2. Department of Physical Chemistry, School of Chemistry, Tel Aviv University, Tel Aviv, Israel
3. Sagol School of Neuroscience, Tel Aviv University, Tel Aviv-Yafo, Israel.
4. Center for Molecular Medicine, University Medical Center Utrecht, Utrecht, The Netherlands
5. Faculty of Medicine, Tel Aviv University, Tel Aviv, Israel
6. Felsenstein Medical Research Center, Tel Aviv University, Petach Tikva, Israel
7. Biobank, Department of Pathology, Rabin Medical Center, Petach Tikva, Israel
8. Department of Digital Medical Technologies, Holon Institute of Technology, Holon, Israel
9. Department of Pathology, Beilinson Hospital Institute of Pathology, Petach Tikva, Israel
10. Neuro-Oncology Unit, Davidoff Cancer Center at Rabin Medical Center, Petach Tikva, Israel
11. The Institute of Imaging, Schneider Children’s Medical Center of Israel, Petach Tikva, Israel
12. Division of Pediatric Neurosurgery, Schneider Children’s Medical Center of Israel, Petach Tikva, Israel
13. Department of Neuro-Oncology surgery and radiosurgery, Rabin Medical Center, Petach Tikva, Israel

**^*^These authors contributed equally**: Galina Feinberg-Gorenshtein and Assaf Grunwald

^†^**Corresponding authors** Corresponding authors: Yuval Ebenstein (uv@tauex.tau.ac.il), Assaf Grunwald (assafgru@mail.tau.ac.il), and Yehudit Birger (yehuditbi1@clalit.org.il)

**FFPE effect on methylation**

To investigate the observed variability in global methylation reduction across FFPE samples, we examined whether the extent of methylation loss correlated with the duration of formalin fixation prior to paraffin embedding. Although our pathology department does not routinely record precise fixation times—available records generally report duration in full-day increments—we observed a consistent trend: longer formalin fixation times were associated with greater methylation reduction compared to matched fresh-frozen (FF) samples (Figure 2c). In contrast, we found no such association with the duration of storage in paraffin blocks (SI table 2). This distinction is particularly relevant for retrospective studies using archival tissue. Based on this trend, we recommend exercising caution when interpreting methylation data from samples fixed for more than 3-4 days.

A plausible biochemical explanation for this time-dependent methylation loss is formaldehyde-induced chemical deamination. Formalin can react with cytosine and 5-methylcytosine (5mC), converting them into uracil and thymine, respectively(1, 2). Given that 5mC deaminates more readily than unmodified cytosine(1), methylated CpGs may be disproportionately affected, leading to an underestimation of methylation levels. In addition, 5-hydroxymethylcytosine (5hmC) may undergo conversion to 5-hydroxymethyluracil (5hmU), a lesion not reliably interpreted by current nanopore basecalling models(3). These chemical modifications likely account for the underestimation of methylation in FFPE samples and provide a mechanistic basis for the observed time-dependent effect. Future studies with controlled fixation conditions and orthogonal validation approaches for different tissue types are essential to establish evidence-based guidelines.

To assess methylation changes at single-CpG resolution, we computed methylation scores for each CpG site in all samples using *modkit* (v0.1.12, Oxford Nanopore Technologies). A scatter plot of FFPE vs. FF methylation levels for each CpG (SI Figure 1) showed a linear correlation within samples, indicating that while global methylation may be reduced, relative methylation levels across the genome are largely preserved—supporting the continued utility of these data for tumor classification.

For this analysis, only CpG sites with coverage ≥5× were included, filtered using AWK and *bedtools intersect* (v2.30.0). Linear regression was performed using Python (v3.10.7) and NumPy (v1.32.2) with the *polyfit* function. Outliers were identified by computing the inverse covariance matrix of the two variables, calculating Mahalanobis distances, and marking values with a chi-square probability >0.65 in red (SI Figure 1).

**Sequence Context and Methylation Loss**

To investigate whether local sequence context influences methylation changes induced by FFPE processing, we stratified CpG sites by their surrounding 4-mer sequence context—defined by the two bases flanking each side of the CpG site (e.g., ACGA, ACGC)—resulting in 16 canonical combinations. For each sample, we calculated the average methylation level per 4-mer group (Supplementary Figure 5).

We then applied a linear mixed-effects model to assess whether methylation loss (Δ = FF − FFPE) was associated with 4-mer sequence context, treating 4-mer as a fixed effect and patient ID as a random effect. ANOVA revealed a significant effect of sequence context (p = 0.0002), with an estimated η² of 0.30, indicating that approximately 30% of the variance in methylation loss could be attributed to sequence differences.

To better understand this effect, we compared how much of the variance in methylation levels could be explained by 4-mer sequence context in two settings: baseline methylation levels in FF samples and the observed methylation loss. A linear model using 4-mer context explained 52.4% of the variance in FF methylation levels (R² = 0.524), whereas in the Δ values, the same model explained only 30.0% of the variance (R² = 0.300). Bootstrap analysis (1,000 iterations) supported the robustness of this difference (mean ΔR² = 0.441, 95% CI: [0.263, 0.602]).

These findings suggest that although sequence context contributes to methylation loss, the majority of the sequence-associated variance originates from pre-existing methylation patterns in untreated FF samples. This supports the conclusion that FFPE processing does not introduce substantial sequence-specific biases, in agreement with the main text summary.

**Impact of FFPE Processing on ONT Sequencing Yield**

To assess whether FFPE-derived DNA results in reduced sequencing efficiency compared to fresh frozen (FF) samples, we performed a time-resolved analysis of sequencing output for patient Sch.1. This pair was selected because both samples were sequenced using the same DNA input amount (SI table 3), thereby minimizing confounding factors. We analyzed the accumulation of sequencing reads and bp over. As shown in Supplementary Figure 2, both the number of reads and the total estimated base yield were lower in the FFPE sample relative to the FF sample.

This analysis is restricted to the first hour of sequencing, which represents the typical time window required to achieve confident methylation-based tumor classification using our assay. The results support the conclusion that FFPE samples may yield reduced sequencing output even under matched experimental conditions, likely due to lower sequencing yield of ONT of fragmented DNA(4).

**CNV PLOTS**

Copy number variation (CNV) plots are a common method for visualizing genomic regions with abnormal DNA copy numbers—either gains or losses relative to the normal diploid state. These alterations are frequently associated with specific tumor types and provide important genetic information that can help guide clinical decision-making.

To demonstrate that CNV profiles can be reliably generated from FFPE-derived DNA sequenced using Oxford Nanopore Technology (ONT), we used the same sequencing data employed during tumor classification sequencing experimet. Supplementary Figure 4 presents CNV plots generated using the ichorCNA tool(5) for two samples that also had CNV profiles available from an orthogonal method. The comparison between the two approaches shows a high degree of concordance, supporting the utility of FFPE ONT data for CNV analysis.

**References**

1. Shen,J. cheng, Rideout,W.M. and Jones,P.A. (1994) The rate of hydrolytic deamination of 5-methylcytosine in double-stranded DNA. *Nucleic Acids Res.*, **22**, 972–976.

2. Privat,E. and Sowers,L.C. (1996) Photochemical deamination and demethylation of 5-methylcytosine. *Chem. Res. Toxicol.*, **9**, 745–750.

3. Xie,N. Bin, Wang,M., Chen,W., Ji,T.T., Guo,X., Gang,F.Y., Wang,Y.F., Feng,Y.Q., Liang,Y., Ci,W., *et al.* (2023) Whole-Genome Sequencing of 5-Hydroxymethylcytosine at Base Resolution by Bisulfite-Free Single-Step Deamination with Engineered Cytosine Deaminase. *ACS Cent. Sci.*, **9**, 2315–2325.

4. Žemaitis,L., Palepšienė,R., Juzėnas,S., Alzbutas,G., Burgi,P.Y., Heinis,T., Charmet,J., Suter,S.A., Jost,M., Raišutis,R., *et al.* (2025) High-performance protocol for ultra-short DNA sequencing using Oxford Nanopore Technology (ONT). *PLoS One*, **20**, 1–10.

5. Adalsteinsson,V.A., Ha,G., Freeman,S.S., Choudhury,A.D., Stover,D.G., Parsons,H.A., Gydush,G., Reed,S.C., Rotem,D., Rhoades,J., *et al.* (2017) Scalable whole-exome sequencing of cell-free DNA reveals high concordance with metastatic tumors. *Nat. Commun.*, **8**.


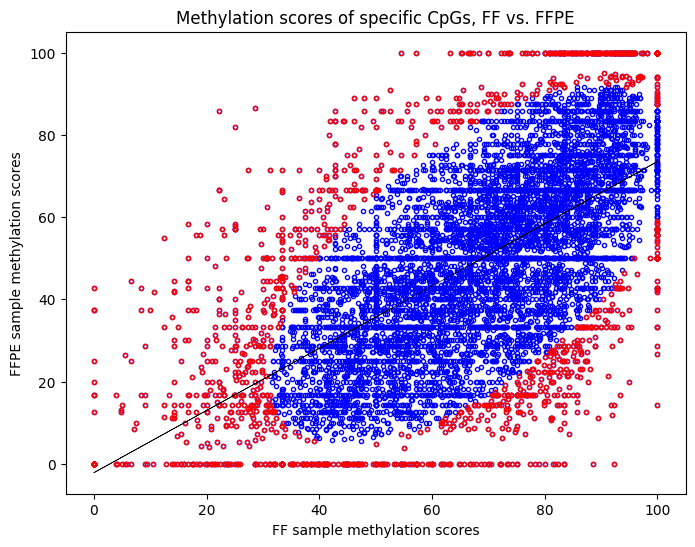


***SI Figure 1****. Methylation values of individual CpG sites in FFPE vs FF samples*

*Methylation values at specific CpGs in FF samples (X-axis) are plotted against these values in FFPE samples (Y-axis). Outliers are colored in red. This plot includes CpG values of all eight patients with coverage > 5.*


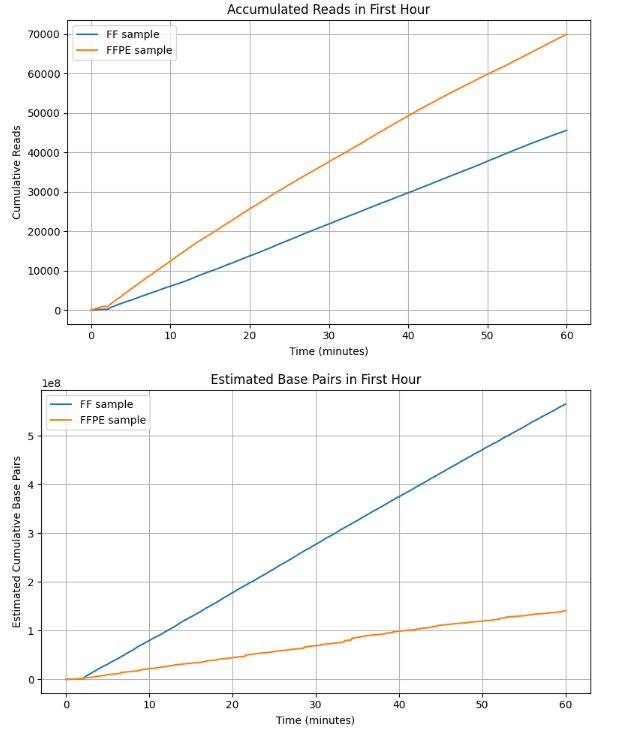


**Figure 2.** *Sequencing efficiency in FF and FFPE samples from patient Sch.1. Both samples were sequenced using identical DNA input amounts . Cumulative number of reads (top panel) and of bases (bottom panel) are shown over the first hour of sequencing.*

*The FFPE sample exhibited higher number of reads, as expected, but lower data accumulation, suggesting reduced sequencing efficiency relative to the FF sample.*


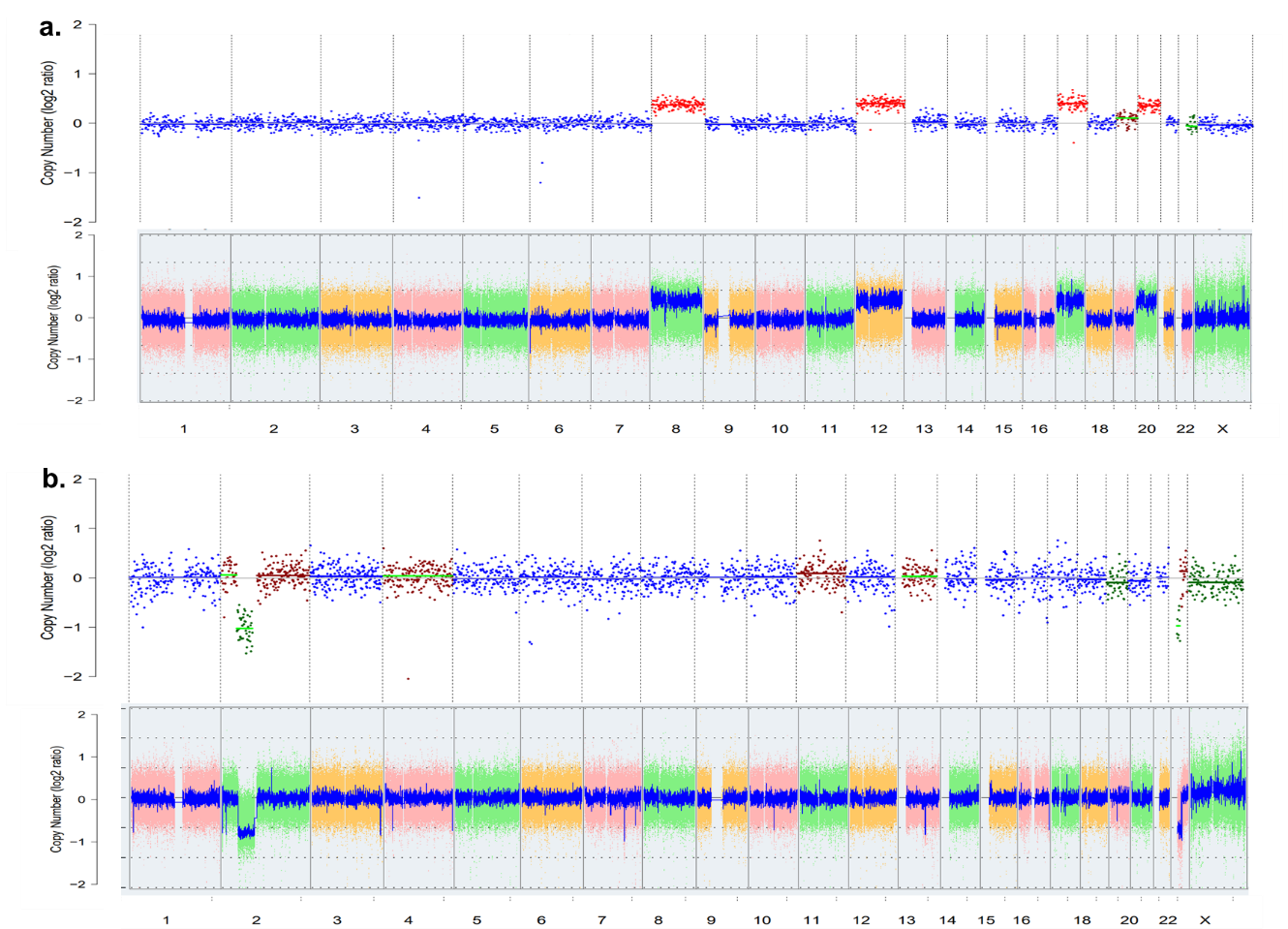


***SI Figure 3.*** Copy number variation (CNV) plots for two patients.

***a****. Patient SCH.10: The upper panel displays the CNV plot generated using our data and analyzed with ichorCNA. The lower panel shows the CNV plot obtained from Chromosomal Microarray Analysis (CMA) (Cytoscan HD, Thermo Fisher Scientific).* ***b.*** *Patient SCH.1: Similarly, the upper panel presents the CNV plot generated from our data using ichorCNA, while the lower panel shows the CNV plot derived from*

***b****.The plots demonstrate a high degree of similarity between the two methods.*


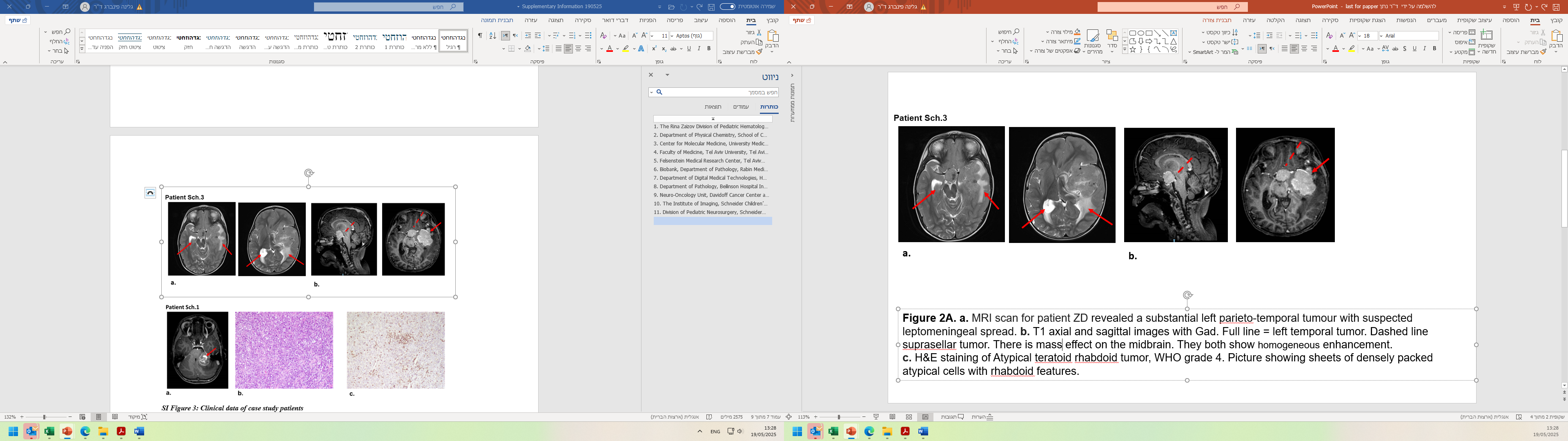

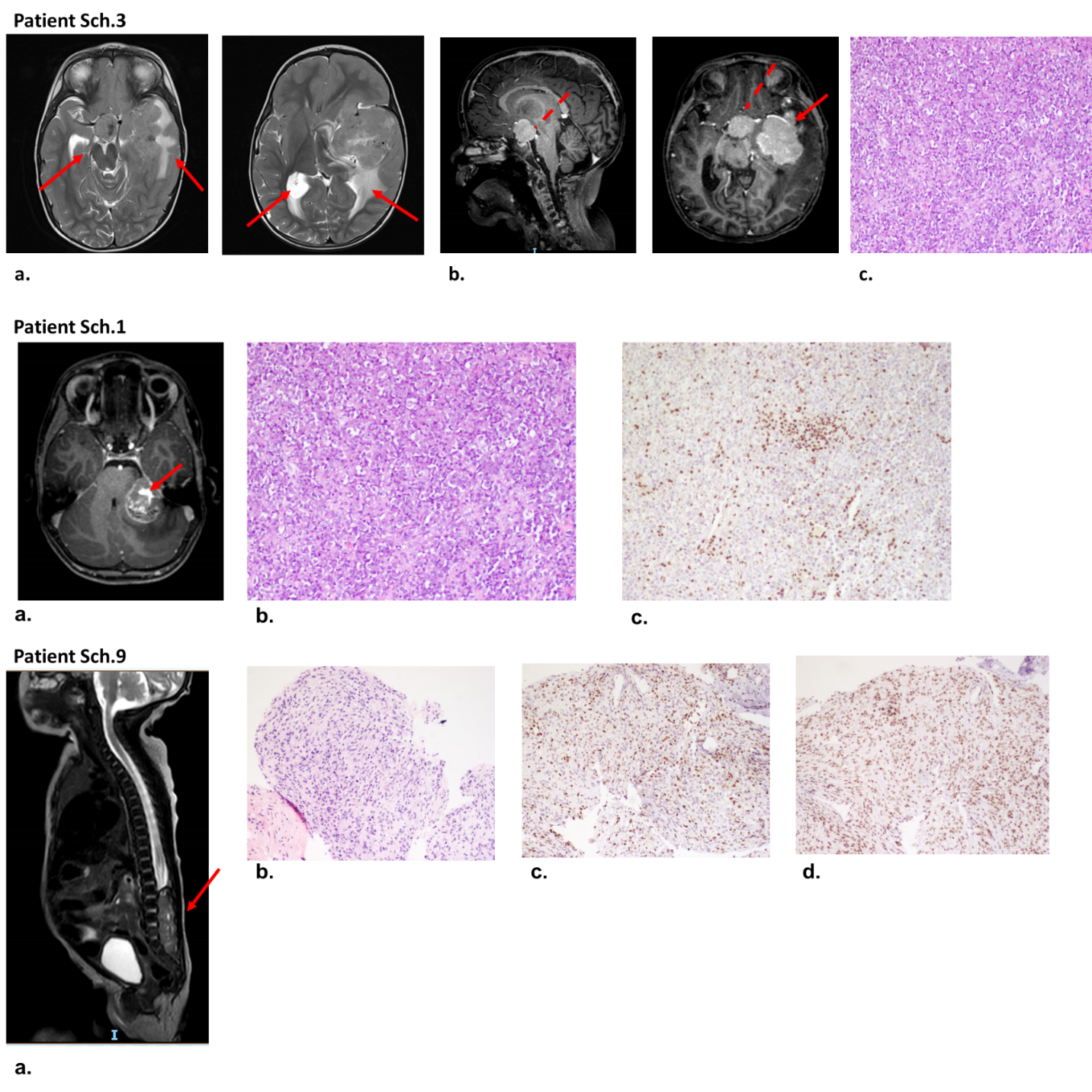


***SI Figure 4.*** *Clinical data of case study patients*

***Patient Sch.3. a.*** *MRI revealed a substantial left parietal-temporal tumor with suspected leptomeningeal spread.* ***b.*** *T1 axial and sagittal images with Gad. Full line- left temporal tumor. Dashed line- suprasellar tumor. Radiolograms revealed a mass effect on the midbrain, and both showed homogeneous enhancement.*

***Patient Sch.1: a.*** *MRI scan shows a tumor in the left cerebellopontine angle with displacement of the 4^th^ ventricle, T1 axial with Gadolinium. The tumor shows heterogeneous enhancement.*

***b.*** *H&E staining****. c.*** *Histochemical staining performed by a pathologist on the tumor biopsy strongly suggested ATRT, but staining for INI1 (SMARCB1), essential for this classification, showed a mosaic pattern.*


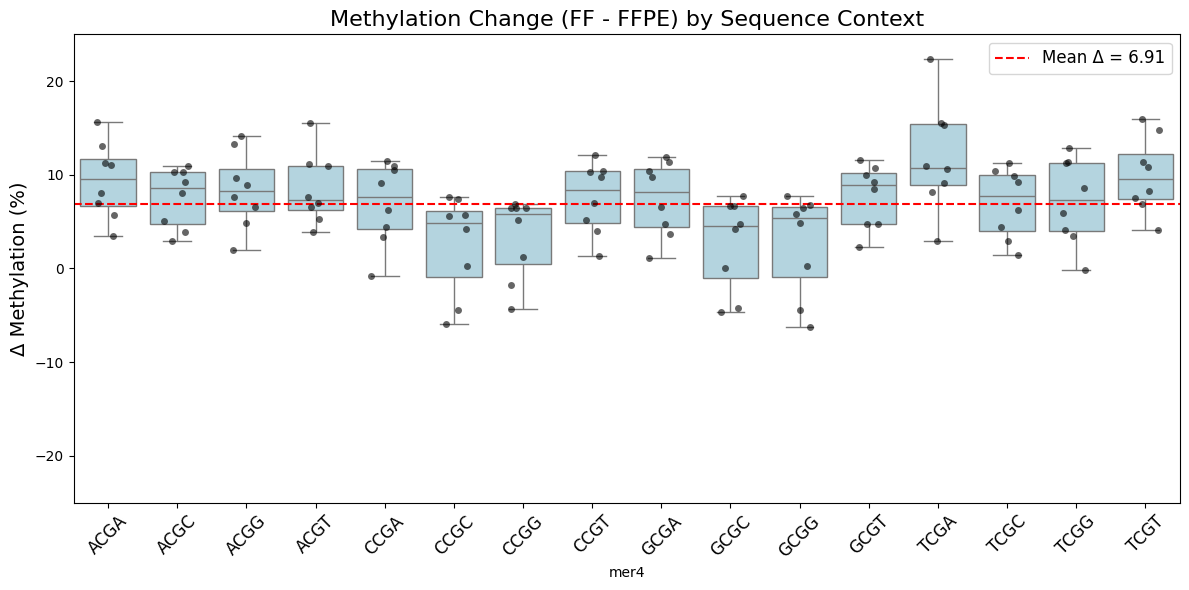


***SI Figure 5.*** *Distribution of Δ-methylation (FF − FFPE, %) across the 16 possible 4-mer sequence contexts flanking each CpG site. Boxplots show the median, interquartile range, and 1.5 × IQR whiskers; dots represent Δ values for each patient within a given 4-mer. The horizontal red dashed line marks the overall mean Δ (6.9 %).*

| **Patient ID** | **DNA Quantity (ng)** | **DNA Quality (260/280)** | **DNA Quality (260/230)** | **No. of probes** | **Significance Score** | **Classification** |
| --- | --- | --- | --- | --- | --- | --- |
| Sch.6 | 1000 | 1.8 | 2.08 | 953 | 0.93 | EPN-PF A |
| Sch.6 | 100 | 1.8 | 2.08 | 2347 | 0.99 | EPN-PF A |
| Sch.6 | 50 | 1.8 | 2.08 | 1932 | 0.99 | EPN-PF A |
| Sch.6 | 25 | 1.8 | 2.08 | 1749 | 0.93 | EPN-PF A |
| Sch.3 | 800 | 1.7 | 1.05 | 15647 | 0.97 | MNG |
| Sch.3 | 100 | 1.7 | 1.05 | 4071 | 0.97 | MNG |
| Sch.3 | 50 | 1.7 | 1.05 | 4667 | 0.97 | MNG |
| Sch.3 | 25 | 1.7 | 1.05 | 1430 | 0.96 | MNG |
| RAB1 | 1000 | 1.8 | 1.9 | 15021 | 0.19 | MB-SHH-A |
| RAB1 | 300 | 1.8 | 1.9 | 4828 | 0.234 | MB-SHH-A |
| RAB1 | 100 | 1.8 | 1.9 | 3311 | 0.228 | MB-SHH-A |

***SI Table 1. Determination of minimal DNA amount required for classification.***

*DNA from three patients was divided into 3-4 sub-samples of decreasing DNA amounts, as detailed in the table. Independent sequencing experiments and classification were performed with each group. Classification scores and the number of probes used for classification (see methods) are detailed for each experimental sub-sample.*
